# Supplementary material for: Reduced social distancing early in the COVID-19 pandemic is associated with antisocial behaviors in an online United States sample
Source: PLoS One. 2021 Jan 7;16(1):e0244974. doi: 10.1371/journal.pone.0244974 (PMC7790541; doi:10.1371/journal.pone.0244974)
Supplement: S1 File — (PDF) [file pone.0244974.s001.pdf]

Supporting Information

Reduced social distancing early in the COVID-19 pandemic is associated with antisocial behaviors in an online United States sample

Katherine O'Connell*, Kathryn Berluti, Shawn A. Rhoads, Abigail A. Marsh

Supporting Text

We used human inspection and an online tool (Prims, Sisso & Bai, 2018; https://itaysisso.shinyapps.io/Bots) to flag and exclude 32 suspicious responses from our dataset. To ensure these methods had no impact on results, we also performed the main analyses when not excluding any suspicious responses (see S1 and S2 Table) and when using a more lenient exclusion criteria (see S3 and S4 Table). For the more lenient criteria, we excluded 12 responses that had the same latitude/longitude information, were flagged by the online tool, and appeared to have free response answers that were bot generated. Varying these exclusion criteria had no impact on the results of the main paper.

S1 Table. Negative binomial regression predicting the number of times participants left their home in the past week, not excluding suspicious responses.

	IRR	95% CI	p-value
Constant	2.575	1.822, 3.638	
Age	1.007	0.993, 1.022	.346
Sex	0.994	0.730, 1.352	.968
Education	0.727	0.498, 1.062	.099
Household income	1.032	0.932, 1.143	.546
Left for work	1.870**	1.288, 2.714	.001
High-risk	0.891	0.643, 1.236	.491
Antisocial behavior	1.007*	1.002, 1.013	.011

n=145, $\chi^2(7)=29.70$, $p<.001$. *p<.05, **p<.01, ***p<.001

S2 Table. Multiple linear regression predicting distance in pixels, not excluding suspicious responses.

	B	95% CI	p-value
Constant	339.130	278.569, 399.690	
Age	-0.274	-2.337, 1.789	.793
Sex	21.179	-21.848, 64.206	.332
Education	-75.277**	-124.292, -26.262	.003
Household income	15.107*	1.650, 28.564	.028
High-risk	8.746	-35.895, 53.388	.699
PPE use frequency	19.951**	6.415, 33.487	.004
Antisocial behavior	-2.040***	-2.862, -1.219	<.001

n=135, $F(7,127)=7.89$, $R^2=.303$, $p<.001$. *p<.05, **p<.01, ***p<.001

S3 Table. Negative binomial regression predicting the number of times participants left their home in the past week, using lenient exclusion criteria for suspicious responses.

	IRR	95% CI	p-value
Constant	2.290	1.303, 4.025	
Age	1.011	0.996, 1.027	.140
Sex	0.940	0.681, 1.298	.708
Education	0.729	0.499, 1.066	.103
Household income	1.027	0.926, 1.138	.614
Left for work	1.717^{**}	1.169, 2.521	.006
High-risk	0.881	0.633, 1.227	.455
Antisocial behavior	1.008^{**}	1.002, 1.013	.007

n=134, $\chi^2(7)=25.18$, $p<.001$. $^{*}p<.05$, $^{**}p<.01$, $^{***}p<.001$

S4 Table. Multiple linear regression predicting distance in pixels, using lenient exclusion criteria for suspicious responses.

	B	95% CI	p-value
Constant	350.063	289.379, 410.747	
Age	-0.337	-2.462, 1.788	.754
Sex	22.524	-21.463, 66.511	.313
Education	-71.204^{**}	-120.698, -21.710	.005
Household income	13.568	-0.001, 27.136	.050
High-risk	16.907	-28.520, 62.335	.463
PPE use frequency	15.912^{*}	2.116, 29.707	.024
Antisocial behavior	-1.753^{***}	-2.604, -0.902	<.001

n=127, $F(7,119)=5.60$, $R^2=.248$, $p<.001$. $^{*}p<.05$, $^{**}p<.01$, $^{***}p<.001$

S5 Table. Pre-registered negative binomial regression predicting the number of times participants left their home in the past week.

	IRR	95% CI	p
Constant	2.195	1.659, 2.905	
Age	1.012	0.996, 1.029	.137
Sex	0.913	0.641, 1.301	.616
Left for work	1.597*	1.051, 2.427	.028
High-risk	0.899	0.632, 1.277	.551
Antisocial behavior	1.006*	1.0002, 1.012	.041

n=117, $\chi^2(5)=17.99$, p=.003. *p<.05, **p<.01, ***p<.001

S6 Table. Pre-registered multiple linear regression predicting distance in pixels.

	B	95% CI	p
Constant	308.168	255.624, 360.711	
Age	-0.674	-2.899, 1.552	.550
Sex	30.880	-15.789, 77.550	.192
High-risk	35.779	-12.024, 83.583	.141
PPE use frequency	16.354*	2.033, 30.674	.026
Antisocial behavior	-2.038***	-2.968, -1.108	<.001

n=114, F(5,108)=5.34, R^2=.198, p=<.001. *p<.05, **p<.01, ***p<.001

S7 Table. Pre-registered multiple linear regression predicting worry about COVID-19

	B	95% CI	p
Constant	3.30	3.01, 3.59	
Age	-0.003	-0.02, 0.02	.771
Sex	0.21	-0.19, 0.61	.300
High-risk	0.55**	0.15, 0.96	.008
Antisocial behavior	0.01	-0.002, 0.01	.154

n=123, F(4,118)=3.17, R^2=.097, p=.016. *p<.05, **p<.01, ***p<.001

Table S8. Negative binomial regression predicting the number of times participants left their home in the past week – with modified STAB-total removing three economic/financial items.

	Step 1			Step 2			Step 3		
	IRR	95% CI	p	IRR	95% CI	p	IRR	95% CI	p
Constant	2.679	2.005, 3.579		2.207	1.587, 3.069		2.563	1.808, 3.634	
Age	1.011	0.995, 1.027	.168	1.013	0.997, 1.030	.111	1.013	0.997, 1.030	.105
Sex	0.724	0.506, 1.036	.077	0.924	0.642, 1.330	.671	0.926	0.648, 1.324	.675
Education	0.992	0.692, 1.421	.964	0.848	0.584, 1.231	.385	0.729	0.493, 1.076	.112
Household income	0.947	0.853, 1.050	.301	0.958	0.861, 1.066	.432	1.002	0.896, 1.120	.974
Left for work				2.029***	1.376, 2.991	<.001	1.659*	1.091, 2.524	.018
High-risk				0.988	0.699, 1.396	.945	0.898	0.633, 1.275	.549
STAB-Total (modified)							1.008*	1.001, 1.015	.028

n=116. Step 1: $\chi^2(4)=3.74$, p=.443. Step 2: $\chi^2(6)=16.00$, p=.014; $\Delta\chi^2=12.83$, p=.002. Step 3: $\chi^2(7)=20.75$, p=.004; $\Delta\chi^2=4.84$, p=.028.
$^{*}p<.05$, $^{**}p<.01$, $^{***}p<.001$

Table S9. Multiple linear regression predicting distance in pixels – with modified STAB-total removing three economic/financial items.

	Step 1			Step 2			Step 3		
	B	95% CI	p	B	95% CI	p	B	95% CI	p
Constant	417.414	375.623, 459.205		379.318	319.687, 438.948		347.069	288.089, 406.049	
Age	-0.239	-2.583, 2.105	.840	-0.187	-2.541, 2.168	.875	-0.280	-2.507, 1.946	.803
Sex	46.467	-2.016, 94.950	.060	44.173	-4.242, 92.588	.073	31.000	-15.318, 77.317	.187
Education	-85.229**	-137.226, -33.233	.002	-86.031**	-137.771, -34.291	.001	-66.868**	-116.857, -16.879	.009
Household Income	15.485*	1.028, 29.943	.036	15.542*	1.164, 29.920	.034	10.893	-2.928, 24.713	.121
High-risk				21.907	-27.502, 71.316	.381	32.650	-14.418, 79.718	.172
PPE use frequency				10.924	-3.666, 25.513	.141	16.098*	2.028, 30.169	.025
STAB-Total (modified)							-1.928***	-2.964, -0.892	<.001

n=113; 1 pixel is approximately equivalent to 0.25 inches (0.64 cm). Step 1: $F(4,108)=3.81$, $R^2=.091$, $p=.006$. Step 2: $F(6,106)=3.12$, $R^2=.150$, $p=.008$; $\Delta R^2=.026$, $p=.200$. Step 3: $F(7,105)=4.93$, $R^2=.248$, $p<.001$; $\Delta R^2=.098$, $p<.001$. B represents unstandardized beta coefficients. *p<.05, **p<.01, ***p<.001

Table S10. Intercorrelations among variables – with modified STAB-total removing three economic/financial items.

	1	2	3	4	5	6	7	8	9	10	11	12	13	14
1. Age	-													
2. Sex	.12	-												
3. Education	.13	.05	-											
4. Income	.02	.10	.38***	-										
5. Left for Work	-.16	-.20*	.10	.07	-									
6. High-Risk	.04	.11	.00	.02	-.01	-								
7. PPE use frequency	-.09	.02	.02	.03	-.07	.07	-							
8. Times left house	-.02	-.12	.04	-.03	.34***	-.05	-.11	-						
9. Distance kept	-.05	.16	-.21*	.07	-.32**	.16	.15	-.38***	-					
10. Worry about COVID-19	.05	.14	-.01	-.07	-.10	.31**	.25**	-.17	.16	-				
11. Physical Aggression	-.13	-.12	.04	-.13	.20*	.16	.07	.25**	-.17	.15	-			
12. Social Aggression	-.03	-.07	.04	-.10	.24*	.10	.07	.25**	-.20*	.12	.85***	-		
13. ***Rule Breaking (modified)***	-.12	-.25**	.07	-.14	.35***	.06	.10	.40***	-.29**	.08	.67***	.63***	-	
14. ***STAB-Total (modified)***	-.07	-.10	.01	-.12	.21*	.13	.08	.26**	-.18	.13	.95***	.96***	.68***	-

n=107. All correlations are Spearman rho values. *p<.05, **p<.01, ***p<.05 Bonferroni corrected for 91 comparisons
