# Supplementary material for: Reduced social distancing early in the COVID-19 pandemic is associated with antisocial behaviors in an online United States sample
Source: PLoS One. 2021 Jan 7;16(1):e0244974. doi: 10.1371/journal.pone.0244974 (PMC7790541; doi:10.1371/journal.pone.0244974)
Supplement: Attachment — Submitted filename: OConnell - Response To Reviewers.pdf [file pone.0244974.s003.pdf]

Response to Reviewers' and Editor's Comments:

Author response to the editor:

Thank you for your thorough and thoughtful comments. We believe our revised manuscript has substantially improved and now addresses all potential confounds, especially those related to economic factors by showing: 1) that removal of economic/financial items of the STAB measure did not affect results, 2) that including household income as a covariate does not affect results (while retaining covariates for demographics, education level and leaving the home for work), 3) that including participant-rated expected economic impact of COVID-19, does not affect results, and 4) that including the "left home for work" variable does not affect results relating antisociality to distance kept from others. We additionally explain why including "worry" as a covariate is inconsistent with our pre-registered hypotheses (but also show that including it as a covariate does not affect results).

We clarify a number of additional aspects of the manuscript. We now detail variations from the pre-registration and report analyses exactly as hypothesized in the supplementary material that show no changes to results. Throughout the paper, we also describe in more detail the context of the pandemic at the time of data collection and how this relates to our chosen dependent and independent variables. Most notably, we explain why PPE use frequency was not investigated as a dependent variable. It is important to recall that during the month preceding data collection, the public was urged *not* to purchase face masks because of shortages affecting healthcare workers (Amazon even temporarily prohibited public purchase of some PPE including N95 and surgical masks) and were even told that masks were not essential for preventing disease spread among non-healthcare workers. Although government guidelines were officially reversed to recommend face masks a few days prior to the study, availability was unequal and public messaging was inconsistent. Because of this, we anticipated the relationship between antisociality and PPE would be mostly uninterpretable, potentially because PPE use frequency would reflect a combination of access to resources/higher income, stockpiling against government officials' recommendations, and concern about COVID-19. We therefore chose to focus on behaviors that violated clear and consistent COVID-19 guidelines, which requested people to stay home (stay-at-home orders affected 94% of Americans) and keep at least 6 feet of distance from others. We believe our results

are meaningful in the context of the early phase of the pandemic and have also modified our title to clarify this.

We additionally clarify methods and variable measurements that reviewers noted as unclear or missing. Finally, we edited the manuscript to ensure that the magnitude of effect sizes are accurately contextualized and that results are not overstated. The list of changes and additional specific comments for each reviewer are included below.

List of changes

- Title
    - Edited from: "Reduced social distancing during the COVID-19 pandemic is associated with antisocial behaviors in an online United States sample" to now reflect that the study was conducted *early* in the COVID-19 pandemic: "Reduced social distancing early in the COVID-19 pandemic is associated with antisocial behaviors in an online United States sample"
- Abstract:
    - Statistical significance was updated based on model changes.
- Introduction:
    - Updated information on the deaths caused by the COVID-19 pandemic.
    - Added citation indicating stability of antisociality.
    - Clarification and additional information regarding the prevalence of antisociality.
    - Added description of the COVID-19 pandemic in the U.S. at the time of data collection, including case numbers, closures, stay-at-home policies that affected 94% of the population, and public messaging inconsistencies on PPE. This involved including additional citations.
- Methods
    - Added description and citations regarding the use of MTurk samples.
    - Added information about income, leaving for work, PPE use and STAB scores to Table 1.
    - Included details of survey questions on risk, open-response questions and income.
    - Included description of the economic/financial items of the STAB antisociality measure, which were removed for a post-hoc analysis.
    - Increased clarity of expected versus obtained sample size.
- Results
    - Both models predicting social distancing (Table 2, Table 3) now include household income as a covariate. The statistical results have been updated throughout the text and in the tables and the coding scheme is described. We have also updated the corresponding tables in Supplemental (Tables S1-S4, S8-S9).
    - Added detail of model differences from pre-registration and included supplemental tables with all proposed analyses exactly as stated in the pre-registration (Tables S5-S7).
    - Added significance markers (*) to Tables 2 and 3 and all supplemental tables to improve clarity.

- Figure 2 caption now indicates the effect was modest for the relationship displayed in Fig 2a.
    - Included results for post-hoc analysis that removed potentially confounding economic/financial items from the STAB. Full table results are reported in the Supplemental (Tables S8-S10).
    - Included all regression covariates in the correlation table (Table 4) as requested, not this resulted in n=107 for the table rather than the original n=121. Minor edits to the text reflect this change.
    - Added description of zero-order correlations and that the relationship between STAB-Total and reported distance kept was at trend level.
    - Added that level of worry about COVID-19 had a positive relationship with the high-risk variable.
- Figure
    - Figure 2 now reflects the inclusion of the household income variable in the model.
- Discussion
    - Added an explicit statement that the individuals reporting "flu-like" symptoms is a small sample.
    - Clarified that the example open-response answers were from social distance violators.
    - Reworded findings and implications to ensure results are not overstated and indicate modest effect sizes.
    - Added two new citations for relevant, newly published results (Nowak et al., 2020; Georgiou et al., 2020).
    - Added discussion of the leaving home for work variable, which predicted the number of times individuals left the home.
    - Added discussion of education and PPE use variables predicting reduced distance kept from others.
    - Added description of how our results and post-hoc tests show that the relationship between antisociality and reduced social distancing is not confounded by socioeconomic factors.
    - Added U.S. National census data describing the race, ethnicity and gender for adults aged 18-65.
    - Added limitations: that observed multicollinearity of STAB subscores precluded us from assessing relation of each subscore to social distancing, no political measures were assessed, that the worry scale may have not captured sufficient detail, that the silhouette image had no contextualization or background.
- References
    - An additional 17 references have been added. One citation has been updated to indicate publication (ref. [61]).
- Supplemental
    - Tables (S1-S4) now include household income as a covariate
    - Tables (S5-S7) are new and report the exact pre-registered analyses
    - Tables (S8-S10) are new and report the post-hoc analyses, which excluded three financial/economic items from the STAB.
    - These changes all continue to support the observed relationship between the STAB and social distancing (i.e. no qualitative change in findings).

Reviewer #1:
The current study presents an analysis of 117 participants who completed self-report questionnaires regarding their COVID-19 pandemic-related compliance with social distancing and use of personal protective equipment (PPE, "mask, face shield and/or gloves") in the previous week as well as basic demographic questions and the 32 item Antisocial Behavior Questionnaire (STAB) inquiring about antisocial behaviors across physical and social aggression, as well as rule breaking domains in the past year.

The authors concluded based on regression analyses including totals scores on the STAB and demographic variables that "antisociality predicted reduced social distancing during the COVID-19 pandemic." The study has some strengths, including an interesting, novel approach to assess subjective social distancing and the authors should be commended for attempting to study of interesting variable in a very timely topic.

I have various concerns about the validity of the study's conclusions that I outline below from major to minor below.

1. *An examination of table 2 shows that the predictors for participants leaving their home in past week were "left for work" and "antisocial behavior" (i.e., STAB total score). The fact that "left for work" is as strong a predictor as "antisocial behavior" for leaving home was unaddressed by the authors and ignores a major alternative explanation for results of study: Economics. The authors may argue that because STAB total scores predicted leaving home in last week independently from "left for work" their conclusions stand. However, an examination of Table 4 indicates that "Rule-Breaking" subscale from the STAB was the most strongly related to "Times Left the House" and the only one to relate to "Distance Kept." An examination of the items of the "Rule Breaking" scale from the STAB shows that there are three items that are all labor-related and could be endorsed by many people in their sample due to pandemic associate massive layoffs ("was suspended, expelled, or fired from school or work"; "had trouble keeping a job"; "failed to pay debts"). It would be interesting to see the rates of endorsement of items in the "Rule Breaking" subscale and see if after removing these 3 items from analyses the results would stand. It is very possible that the observed results for the "leaving home" outcome are driven by people who must leave for work because they are "essential" either in the higher end of education (e.g., medical personnel) but also in the lower end (cashiers, truckers, agricultural workers) who are also the most vulnerable to losing their jobs in the past year and thus endorse those 3 "Rule Breaking" items that basically penalize economic vicissitudes.*

We appreciate the reviewer for raising these important points related to economic impacts and the STAB measure. We removed these three economic-related items from the STAB and now report brief results in the main text (lines 302-312) and full results in the Supplemental Materials (Table S8-S10). All main findings persisted after removing these items and the correlations between the Rule-Breaking subscale and social distancing measures were similar (rho values increased slightly; see Tables 4 and S10). These results show that economic hardship (which could

result in higher STAB-Total and STAB-Rule Breaking scores) did not underpin the main findings relating antisocial behavior with reduced social distancing. We have modified the manuscript to detail these results (lines 213-216, 302-312, 398-402)

Regarding the results in Table 2, leaving home for work is indeed a significant predictor for times left the house in the past week as expected. However, because STAB-Total remains a significant predictor in the model after accounting for this work variable, we suggest that antisociality acts as an independent predictor. We have added discussion regarding these findings to the discussion section (lines 393-398). In addition, to ensure there was no interaction between STAB-Total and leaving home for work, we tested the same regression model (Table 2), but included an interaction term between these variables. We found no trending or significant effect for this interaction term (IRR=.998, 95%CI =[0.987, 1.010], p=.742; we observed a similar result when using the modified STAB-Total score removing economic items p=.795).

We next assessed responses to an item not previously discussed in the manuscript: "to what extent do you think you will be impacted by the financial/economic outcomes of COVID-19 (for example from economic recession or job loss)", which was rated on a 5-point scale from "Not at all"=1 to "Substantially"=5. Hypotheses related to this question were beyond the scope of the current paper; however we note: **1)** there was no relationship between responses to this question and times left the home (rho=-0.06, p=.520, n=131), **2)** there was no difference between financial impact ratings of participants who left for work versus those who did not leave for work (left for work: M=3.34, SD=0.94, n=29; did not leave for work: M=3.36, SD=1.11, n=95; t(122)=0.05, p=.954), and **3)** when we included this financial impact variable as a continuous measure in Step 3 of the model (Table 2, Step 3) STAB-Total score remained a significant independent predictor (IRR=1.009, 95%CI=[1.003,1.015], p=.004; we observed a similar result when using the modified STAB-Total score removing economic items p=.005) for times leaving the house in the past week. We believe these results support that our main findings were not driven by economic impacts of COVID-19 or confounded by economic disparities between participants. We believe that data related to the expected financial impact variable were beyond the scope of the current paper and therefore have not incorporated these results into the manuscript, but these data remain available in the public dataset.

2. *My hypothesis above is supported by the authors' own data. Table 3 shows that less self-reported distance from others is predicted by higher education and more PPE use. In*

short, possibly by people who are essential workers in higher and lower SES strata who by the nature of their work (physician, cashier) have to leave home AND keep less distance from others. Again, the fact that higher education and PPE use predicts lower distance is glossed over and not addressed respectively in the discussion of the article, and supports the possibility that what we are seeing is the grim reality of the pandemic: Essential workers are having to put themselves at higher risk and the way they cope with is through increased PPE use. Those who have to leave home and be closer to others may do it because they have been "fired from work" and thus "had trouble keeping a job" and consequently are have "failed to pay debts." Certainly not antisociality.

We have added more discussion of PPE use and education as predictors of distance kept to the discussion (lines 407-425). Note we found the opposite of what is stated above: more frequent PPE use was associated with *increased* distance kept. While it is difficult to say exactly why higher education was associated with reduced distance kept, we do not believe these results are related to healthcare professionals working in close proximity to others, especially since only 1 participant reported working as a healthcare professional (line 148). We tested the model (Table 3, Step 3) including the "left home for work" variable, and observed this variable does act as a significant predictor of reduced distance kept from others (B=-74.39, 95%CI =[-139.08, -9.69], p=.025), indicating that subjects who were working outside of the home reported keeping less distance from others. Importantly however, after adjusting for this work variable, the relationship between increased STAB-Total and reduced reported distance persisted (B=-1.47, 95%CI =[-2.47, -0.48], p=.004), again indicating the relationship between STAB-Total and violations of social distancing is not driven by economic factors.

3. *My hypothesis is further supported also by the authors' own report that none of the STAB subscales or total score were related to PPE use. This further suggests that the reported relationship between "antisociality" and pandemic social distancing norms that the authors report have little to do with flaunting of social norms (mask wearing being one of the most contentious pandemic norms in the U.S.) and more with financial need to go to work. If antisociality assessed by the STAB which includes hitting, threatening, and swearing indeed was affecting these norms, some relation to PPE (arguably an easier to break norm) would be observed but that is not the case.*

Because data was collected at an early phase of the pandemic, PPE was not easily accessible to Americans and messaging from public health officials was inconsistent, often focusing on reserving PPE for healthcare workers rather than public use. For example, the U.S. Surgeon General Twitter account posted, "**Seriously people - STOP BUYING MASKS! They are NOT effective in preventing general public from catching #Coronavirus…**" on February 29th 2020

and only reversed this messaging on April 3rd 2020, which was 4 days prior to data collection (now cited in the manuscript; lines 92-94, 417-419). In the weeks immediately preceding our study's data collection, two primary social distancing guidelines were clear and consistent: physical distancing of at least 6 feet, and official recommendations to stay at home. Our a priori hypotheses focused on these behaviors that were clear violations of COVID-19 guidelines at the time. Especially because 40.0% of respondents reported never or rarely using PPE in the past week, we suggest extreme caution when drawing any conclusion from this variable as it may simply reflect access to PPE at this particular time point (or even may have been indicative of stockpiling behavior, which could complicate an expected relationship with antisociality as shown by recent research; Nowak et al., 2020). We added thorough discussion of why we did not assess PPE as a dependent variable to the manuscript (lines 92-94, 417-419).

As we show in prior responses, economic factors and participants' need to leave for work are important variables that we take into consideration, but we show that the relationships between STAB-Total score and violations of COVID-19 social distancing guidelines persists after accounting for these variables.

4. *Incidentally, the data suggests that the contentiousness of social distancing and mask-wearing (or not) in the U.S. breaks along party/ideological identification with more democrat/liberal individuals endorsing more mask wearing whereas more republican/conservatives endorse less mask wearing (Pew Report, 2020). I don't know if the authors asked any data in this respect but in general, republican/conservative individuals tend to endorse more traditional, norm following attitudes. Those data would militate against "antisocial behavior" as an explanation for failure to maintain social distance or remaining at home. The possibility that blue-collar jobs that are being threatened in this economy may be held by persons with more conservative ideology could however be playing a role. Or that more conservative leaders are disavowing social distancing and mask wearing citing economic reasons. Indeed, some of the qualitative data mentioned by the autors in p.18 suggest that economic anxiety is at the core of some of their participants' behavior ("I worry that my retirement accounts won't recover" "…cannot move ahead in life [sic]to the economy gets going again…"If the authors had a variable to address the influence of political ideology on COVID-19 related health norms would strengthen their study significantly. Alternatively, (although I am often reluctant to be the reviewer who says 'collect more data') doing another rapid survey on MTurk as the authors did, with a larger sample that assesses political ideology, recent loss of job because of the pandemic and economic stress due to it, and controls for it in their models could provide far more illuminating results.*

While we agree that political ideology would be an interesting variable to assess both for social distancing and economic/ worry ratings, we do not have this information. Due to the rapidly evolving politicized nature of the pandemic over time, we do not believe that additional data collection now would help explain results from early April. However, we have added a reference to a relevant pre-print that indicated political conservatism is associated with reduced reported social distancing, i.e. less avoidance of social gatherings and not keeping 6 feet from others (lines 439-443).

5. *An important variable related to the above concerns also not addressed in the analysis is the effect of ethnicity in their findings. The pandemic has hit lower SES workers and in especially Black and Latino workers and communities. The numbers of individuals from these communities are underrepresented in the study's sample (perhaps another reason to continue collecting data and oversampling these populations) but nonetheless, it would behoove the authors to explore the interaction between these two ethnicities and endorsement of the three labor related items from the "Rule Breaking" scale of the STAB. The results may show yet another grim reality from the pandemic: The endorsement of having to leave home more often and be closer to other individuals is not due to "antisociality" but due to Black and Latino households having a higher rates of job losses during the pandemic, higher rates of burden for the caring of more family members, less work flexibility, and increased need to find more dangerous jobs during a pandemic.*

Our sample included 11.5% subjects who identify as Hispanic or Latino, and 19.8% who identify as Black or Latino. Consistent with the reviewer's suggestions, Black or Latino subjects more frequently report leaving home for work (58% relative to 17% of non-Black or Latino subjects, χ^2=21.6, p<.001) and had higher scores on the economic items of the STAB (Black/Latino: M=8.23, SD=0.79; Not Black/Latino: M=4.03, SD=0.21; t(129)=7.36, p<.001). However, as we describe above, we show that the relationship between antisociality and reduced social distancing persists after: 1) removing economic items of the STAB-Total, and 2) adjusting for leaving for work, education and household income. The pandemic absolutely is a burden on lower SES workers and minority communities, although epidemiological evidence shows this also is a *notable change* since the earliest days of the pandemic; see, for example, a March 14 NPR story titled, "COVID-19's Global Spread Among The Relatively Rich Has Been Remarkable". We now discuss this and clarify that our findings should not be interpreted to suggest that going to work is an antisocial behavior (lines 397-398).

6. *Given the above concerns, the authors should explain why they chose to use the total score of the STAB-Total score in the regression analyses given that most of the variance is accounted for by the "rule breaking" scale. As I mentioned, I suspect that their results are better explained by endorsement of the 3 labor-related items in it and related to pandemic related massive layoffs rather than frank rule breaking. However, even if after removing these items, the (relatively) weak effect results hold, the authors would have to find a way to better contextualize their results. Otherwise they could be summarized as "people who do not follow strong social norms of behavior like stealing, and destroying private and public property are more likely to leave the home (paradoxically, possibly to work) and keep slightly less distance from others during a pandemic." To be fair, the authors should be commended for trying to study such a fluid and novel phenomenon. However, the sample size, measures and thoroughness of analyses compares unfavorably with other efforts that are emerging with similar topic (e.g., Georgiou et al., 2020; Nowak et al., 2020).*

We have added these two citations to our discussion to help contextualize our results (lines 381-385; 387-388). Our study implemented a standard measure of antisociality with predictive validity for violent crimes, criminal convictions, and acting-out in community samples. Our results show that, even after adjusting for socioeconomic variables as the reviewer suggests, STAB-Total score acts as an independent predictor of reduced social distancing during an early phase of the pandemic. We believe the supplemental analyses removing economic items from the STAB scores increase confidence in our findings and thank the reviewer for suggesting them. The magnitude of effect sizes are now more explicitly noted (Figure 2 caption, lines 367, 403-407), but we note the sample size was determined to be sufficient based on a pre-registered power analysis. Our study had a relatively narrow focus specifically on antisociality, but larger future studies could more deeply query related factors such as political ideology and racial/ethnic inequalities.

References

Bartłomiej Nowak, Paweł Brzóska, Jarosław Piotrowski, Constantine Sedikides, Magdalena Żemojtel-Piotrowska, Peter K. Jonason, Adaptive and maladaptive behavior during the COVID-19 pandemic: The roles of Dark Triad traits, collective narcissism, and health beliefs, Personality and Individual Differences, Volume 167, 2020, 110232, ISSN 0191-8869, https://doi.org/10.1016/j.paid.2020.110232.

Neophytos Georgiou, Paul Delfabbro, Ryan Balzan, COVID-19-related conspiracy beliefs and their relationship with perceived stress and pre-existing conspiracy beliefs, Personality and Individual Differences, Volume 166, 2020, 110201, ISSN 0191-8869, https://doi.org/10.1016/j.paid.2020.110201.

"Republicans, Democrats Move Even Further Apart in Coronavirus Concerns." Pew Research Center - U.S. Politics & Policy, Pew Research Center, 24 July 2020. www.pewresearch.org/politics/2020/06/25/republicans-democrats-move-even-further-apart-in-coronavirus-concerns/.

Reviewer #2:
This report uses data collected from 131 MTurk workers to evaluate the associations between antisociality and three variables related to feelings and behaviors during the Covid-19 pandemic in the Spring of 2020: the number of times participants left their homes during the past week, an estimation of typical social distancing, and worry about the pandemic.

Reviewer background: I read the paper and the pre-registration. I did not check the analyses myself as I do not use STATA.

Strengths: The topic is interesting and timely. The work was pre-registered. The report was efficiently written. Count regression models were used for the count DV. I think the main result is interesting in that it suggested (at least to me) an association between general antisociality and certain ways of acting in a pandemic that are harmful to others. The social distancing measure was novel.

Weaknesses: Cross-sectional data, sample might be selective, some deviations from the pre-registration. There was a power analysis but as an individual difference researcher, I typically prefer larger samples for more precise estimation of relevant coefficients.

Three Pre-Registration Comments.

1. *The authors pre-registered the following covariates for the models in Tables 2 & 3 = age, sex, use of PPE, high risk group, and occupation requiring one to leave home [omitted for the distance DV]. Tables 2 & 3 also include education. So, education was not pre-registered (as best as I could tell). This should be acknowledged assuming I am not missing something.*

The reviewer is correct. We added education based on early feedback from a colleague and have now added household income based on reviewer feedback. We more clearly acknowledge this in the manuscript (line 258-261), and now have added all pre-registered analyses to the supplemental materials (Tables S5-S7). There are no qualitative differences in results.

2. *The pre-registration also notes a sub-aim to test whether the STAB "effects" are primarily driven by the social aggression subscale. This issue was likely hard to test given that the STAB scales are strongly correlated but those null results could be summarized in the paper with a sentence. (If anything, it looks like the Rule Breaking scale had the largest correlations, but I doubt there would be evidence that that the coefficients differed from one another).*

Because of multicollinearity (observed VIFs>10), we were unable to test for predictive differences among subscores, but instead report the zero-order correlations (Table 4). We included discussion of this to the study limitations and noted our pre-registered hypothesis (lines 435-439)

3. *It should probably be noted that the authors wanted to have a sample of 138 but had to deal with 131 for the first analysis. This is not a deal-breaker at all to me, but I just like to see deviations noted in papers that have pre-registration. (The authors did something savvy as well by setting power to .90 rather than the typical .80 value that often makes little sense.)*

We thank the reviewer for noting this detail. This was briefly described, but is now more clearly indicated in the manuscript (line 227).

General Comments

4. *The zero-order STAB effect for distancing was not $p < .05$ best as I could tell. That might be worth a comment.*

We now specify that the Spearman correlation was at trend level for this relationship on line 334 (rho=-.18, p=.06).

5. *I think adding means and SDs to Table 4 would be useful. I would also add all the variables used in the regression models to this table. For example, left for work and high-risk could be included.*

We thank the reviewer for these suggestions. We have updated Table 4 to include all variables used in the regression models (also to Table S10, which assesses the modified STAB scoring after removing economic factors). To keep with the organization of the paper and to more fully describe the ordinal variables, we have added additional summary statistics to Table 1 for household income, leaving for work, PPE use frequency and STAB scores.

6. *I think the attention checks for MTurk workers were savvy. The authors might want to say more about the use of MTurk workers. I assume that the state data was too sparse to try a multilevel model given that states had different policies.*

We appreciate the reviewer's comment and have added details and references about the use of MTurk workers (lines 139-142). While we did collect geographical information, but this study was not designed to assess geographical differences. However, we now note that by the end of

March, 42 states and additional cities/counties were under stay-at-home orders, which affected 94% of the U.S. population (now cited on lines 85, 422).

7. *The authors could add a few sentences to describe the pandemic conditions in the USA during April 8 and the preceding week.*

We have added details about the COVID-19 conditions in the US to the introduction section (lines 80-86), "Confirmed U.S. cases increased 112% from 186,101 on April 1st to 395,011 on April 8th [29] (the day of data collection), community spread was confirmed to be present in at least 31 U.S. states [30], and general uncertainty regarding risks and illness transmission coincided with widespread complete closures of non-essential businesses such as restaurants."

We also now discuss guidelines related to PPE use and stay-at-home orders to both the introduction (lines 89-97), and discussion (lines 415-425) sections.

8. *The high-risk variable could be described a bit more in the paper.*

Thank you for mentioning this oversight. We have added details about the risk questions and coding scheme to the methods (lines 177-183).

9. *The power analysis used a poisson regression but the negative binomial was reported. The data were likely more over dispersed than ideal for the standard poisson model so the negative binomial is more appropriate and I think they have better type I error control in this case. (I am working from home and my texts are in my office so I might be misremembering these details). Anyways, I don't know what (if anything) this difference in models means for the power analysis.*

We did not expect sample size calculations would meaningfully differ between a Poisson and negative binomial regression, but we notably had no means of estimating overdispersion prior to data collection.

10. *The reported worry single item DV might have more measurement error than ideal for testing the hypothesis. This could be noted as a limitation.*

We added the following limitation to line 441, "We also note that worry about COVID-19 was measured on a 1-5-point scale, which may not have provided sufficiently detailed information to detect a relationship with antisociality."

11. *I think the conclusions on the top of page 19 (esp. the "even a small population of antisocial hosts…") were a bit too strongly worded for my taste.*

These conclusions have been reworded to now say, "through this report, we hope to convey that antisociality serves as a significant, albeit modest, predictor of behaviors relevant to infectious disease spread. Our results, if confirmed and extended, may suggest that even a small population of antisocial hosts could have important implications for the propagation of a global pandemic like COVID-19." (lines 366-369).

12. *I think the Discussion was otherwise interesting and I appreciated the connection to other recent studies.*

We appreciate this comment as we also feel this manuscript helps confirm and extend findings from at least 3 other similar reports.

Reviewer #3:
This manuscript examines whether antisocial behavior is associated with reduced social distancing during the COVID-19 pandemic. This paper addresses a timely question and does a good job of explaining the relevance of the work to public health. However, there are several issues with the conceptualization of the study and the methods/results. These are described below.

Background Section:

1. *The authors discuss antisociality as an underlying phenotype. The citations provided do not provide sufficient support for this argument. The following is an example of an article that would provide much better support for this assertion: Niv, S., Tuvblad, C., Raine, A., & Baker, L. A. (2013). Aggression and rule-breaking: Heratibility and stability of antisocial behavior problems in childhood and adolescence. Journal of Criminal Justice, 41.*

We thank the reviewer for recommending this citation, we now include this reference (line 62).

2. *Generally, antisocial behavior is discussed as a unitary construct. This is problematic, as even one of the articles cited by the authors argues that there is only moderate overlap in genetic influences on aggression and rule-breaking behavior (Burt, 2013). In addition, the authors are sometimes vague as to what they mean by "antisociality." They argue that antisociality predicts violence, based in part on papers that examined antisocial personality disorder in relation to violence. Violence in itself could be considered a manifestation of antisociality. Therefore, it is unclear how the researchers are defining this term.*

We define antisocial behavior as any action that harms others, violates social norms, or infringes on the rights of others (lines 67-68). Although ideally we would have been able to comment more on the subscores of the antisociality measure—which breaks down rule-breaking, physical aggression, and social aggression—high correlations among these scores made this statistically infeasible. Furthermore, we aimed to assess antisociality broadly across the general population, for which the STAB questionnaire was well suited, but future work may conduct more targeted questions related to subtypes of antisocial behavior. We added additional information and clarity to our description of antisociality in the introduction and its association with other health behaviors (lines 70-73)

3. *On page 3, the authors state, "The tendency to engage in antisocial behaviors such as violence, rule-breaking, and bullying varies significantly across the population, with a small proportion of individuals responsible for the majority of antisocial acts." The citations used to justify this statement only apply to serious antisocial acts, rather than less serious antisocial acts that may be more evenly distributed in the population.*

We appreciate this thoughtful comment and now more clearly note this (line 70, 72).

Methods and Results

4. *In the discussion, the authors argue that the sample roughly approximated major demographics of US adults ages 18-65. The authors should provide this demographic data so that the reader can judge for themselves the extent to which the sample was in fact representative.*

We would like to mention that the second half of the sentence in question states that our sample, "should not be considered nationally representative", but we also now include the requested statistics (76% White, 14% Black, 7% Asian; 23% Hispanic; 50% female; national data on adults 18-64 as of 2019) (line 427-428).

5. *The theoretical basis for the classification of variables as predictors, outcome variables, or covariates is unclear. For example, why is PPE use frequency treated as a covariate rather than an outcome? Similarly, it seems that reported worry about COVID-19 might be an important covariate rather than an outcome variable.*

See our response to Reviewer 1, question 3, which addresses these concerns. Namely, our dependent variables for social distancing were chosen because they represented clear and consistent violations of public health guidelines at this early phase of the pandemic. We did not

use PPE use frequency as a dependent variable because PPE and masks were not easily accessible to Americans at the time (Amazon temporarily banned sale of N95 and surgical masks to the U.S. public on April 1), and messaging from public health officials was inconsistent, sometimes focusing on reserving PPE for healthcare workers and downplaying their importance for public health. Therefore, we anticipated the relationship between antisociality and PPE would be mostly uninterpretable, potentially because PPE use frequency would reflect a combination of increased access to resources/higher income, stockpiling against government officials' recommendations, and concern about COVID-19. Obviously, later in the pandemic masks are now seen as a primary method of reducing transmission (although also now show complex politicization), and future work studying different phases of the pandemic should account for this. We now more clearly indicate these points in lines 92-94, 417-419.

We do not use worry about COVID-19 as a covariate, since we expected it might partly explain a relationship between antisociality and social distancing (such that more antisocial individuals would worry less, and relatedly, engage in less social distancing). However, we show below that including worry as a covariate does not meaningfully impact the relationship between antisociality and social distancing. And although it increases the fit of the model predicting times leaving the home, we do not include it as a covariate due to our hypotheses. Both PPE use frequency and worry about COVID-19 were included in the manuscript exactly as stated in the pre-registration.

Modified Table 2. Negative binomial regression predicting the number of times participants left their home in the past week.

	Step 3			Worry Step		
	IRR	95% CI	p	IRR	95% CI	p
Constant	2.578	1.818, 3.657		5.218	2.853, 9.542	
Age	1.013	0.997, 1.030	.105	1.012	0.996, 1.028	.128
Sex	0.925	0.647, 1.322	.669	0.957	0.676, 1.356	.807
Education	0.725	0.491, 1.071	.106	0.735	0.504, 1.072	.110
Household income	1.004	0.897, 1.123	.946	0.996	0.893, 1.111	.947
Left for work	1.650	1.084, 2.510	.019	1.481	0.978, 2.243	.063
High-risk	0.894	0.630, 1.270	.531	1.050	0.732, 1.505	.793
Antisocial behavior	1.007	1.001, 1.013	.024	1.009	1.003, 1.015	.004
**Worry COVID-19**				0.803	0.688, 0.938	.006

n=116. Step 3: $\chi^2(7)=21.00$, p=.004; Worry Step: $\chi^2(8)=28.47$, p<.001; $\Delta\chi^2=67.63$, p=.006.

Modified Table 3. Multiple linear regression predicting distance in pixels.

	Step 3			Worry Step		
	B	95% CI	p	B	95% CI	p
Constant	346.500	287.482, 405.519		317.342	232.815, 401.87	
Age	-0.264	-2.489, 1.961	.815	-0.245	-2.471, 1.982	.828
Sex	31.109	-15.171, 77.389	.185	28.362	-18.291, 75.015	.231
Education	-66.380	-116.389, -16.372	.010	-67.153	-117.213, -17.093	.009
Household Income	10.663	-3.171, 24.497	.129	10.936	-2.917, 24.789	.121
High-risk	33.215	-13.863, 80.293	.165	27.615	-20.898, 76.129	.262
PPE use frequency	16.089	2.029, 30.150	.025	14.537	0.106, 28.968	.048
Antisocial behavior	-1.756	-2.695, -0.817	<.001	-1.787	-2.729, -0.845	<.001
**Worry COVID-19**				10.367	-11.137, 31.872	.341

n=113. Step 3: F(7,105)=4.95, R^2=.248 p<.001; Worry Step: F(8,104)<.001, R^2=.255 p<.001; ΔR^2=.007, p=.341.

6. *Participants came from 38 states. As disease spread varied widely by state in April 2020, including covariates that capture the extent of the state's outbreak, as well as shutdown regulations is important. Income is another important covariate that is not included.*

We now include household income as a covariate in the main models, which is also in line with Reviewer 1's comments. We have highlighted this variable in Tables 2 and 3. No qualitative differences were observed for the relationship between antisociality and social distancing after adjusting for income.

Disease spread did vary at this point, but government issued stay-at-home/safer-at-home orders affected 94% of the U.S. population, which is now indicated in the manuscript (lines 85, 422). While we did collect geographical information, this study was not designed to assess geographical differences. However, we tested the inclusion of county-level total number of COVID-19 cases as of April 1st 2020 as a covariate and found no meaningful differences (information obtained using self-reported ZIP code and CDC COVID-19 county reports). Local COVID-19 cases were not significantly associated with times leaving the home (IRR=1.000, 95% CI=[0.999, 1.000], p=.675), whereas STAB-Total remained a significant independent predictor (IRR=1.007, 95%CI=[1.0003,1.013], p=.041). Local COVID-19 cases also were not significantly associated with distance kept (B=-.008, 95% CI=[-.019, .004], p=.205), but STAB-Total again remained a significant independent predictor (IRR=-1.73, 95%CI=[-2.68, -0.78], p<.001). We chose not to

include this covariate in the manuscript since we are unable to validate the data and there was no way for us to know whether participants were knowledgeable about COVID-19 spread in their county.

7. *The use of the silhouette image to assess social distancing is problematic. The image does not include any background images that might help the participant to gauge the relative size of the image. The authors interpret the image as being an average height male for their calculations, but this might not have been clear to the participant from the image. Also, why do the authors not take into account y-axis information when taking measurements? This would be relevant, as participants themselves had the ability to select distances based on both the x- and y-axes.*

The silhouette image used in our study was novel, although the use of human-shaped avatars for studies assessing social distance-related variables is commonplace, including in our own research (e.g., Vekaria et al 2017, *Nature Human Behaviour*). We now note these concerns in the limitations section, "the silhouette image had no specific contextualization and could be adapted in the future to include, for instance, the backdrop of a grocery store, which may increase measurement precision" (lines 447,449).

We chose not to consider y-axis information because we expect this would increase unmeaningful variance in the data, for example potentially related to participants' height.

8. *Minor Point: Generally, correlation results are presented before regression results.*

While we agree, we prefer to keep the current organization, which is consistent with how the analyses were pre-registered.

9. *The authors acknowledge this, but the lack of data on the reason for leaving home is a serious limitation. It could be the case that antisocial individuals had to leave the home for reasons that were not driven by their antisocial tendencies. Without data on the reasons for leaving home, it is difficult to derive causal inferences.*

We note that we do adjust for participants leaving the home for work, and now clearly specify that during this stage in the pandemic 94% of the U.S. population was advised not to leave their homes for all non-essential purposes. However, we agree this is a limitation as previously mentioned in the discussion (lines 443-447). We have also modified the manuscript to be more cautious in interpretations throughout as mentioned above.

Discussion

10. *The authors state that 9.4% (3/32) of those who reported standing less than 6 feet from others also reported having flu-like symptoms. Given that this sample size is very small, it is not appropriate to draw conclusions or report results from these 3 participants.*

Based on the posited importance of a small (1-10% of the population), but persistently antisocial group of individuals in the context of an infectious disease pandemic, we believe this information is relevant to the reader. We now explicitly mention the small sample (line 355).

11. *Answers to open-ended responses should not be reported in the discussion. These are results that should be reported in the results section and explained in the methods section.*

We added description of the open-ended response question to the methods section (lines 191-197). The data collected for this question is planned to be used in a separate manuscript, and we therefore are only use responses to help discuss and contextualize our findings—which we believe is best done in the Discussion section, although we are open to comments from the editor on further revisions.
