# Supplementary material for: Reduced social distancing early in the COVID-19 pandemic is associated with antisocial behaviors in an online United States sample
Source: PLoS One. 2021 Jan 7;16(1):e0244974. doi: 10.1371/journal.pone.0244974 (PMC7790541; doi:10.1371/journal.pone.0244974)
Supplement: Attachment — Submitted filename: OConnell - Response To Reviewers.pdf [file pone.0244974.s004.pdf]

December 18th, 2020

Authors' responses to the editor:

"Reviewer 1 had a couple of remaining suggestions that would help integrate economic and ethnic/race explanations in the interpretation of results."

Our responses to Reviewer 1 are below.

"1) reduce causal or "predictive" language"

We agree with the importance of using caution when it comes to causal language, and we have edited the manuscript to reduce causal language in the following places: line 348, line 370, line 410, line 470. Relatedly, we edited the abstract to reflect that sociodemographic variables were also associated with social distancing behavior.

"2) make clearer that the MTurk sample doesn't necessarily parallel US demographics, especially around income/education levels"

We agree, and added the following sentence to the discussion section (lines 441-442), "MTurk samples are also not representative of U.S. sociodemographic variables and tend to include individuals with higher education and income [80]"

"3) acknowledge potential personality or other mechanisms that explain associations between antisociality and violations of COVID-related social distancing guidelines (relevant to previous concerns in the first round of reviews about addressing confounds and reducing overattribution of effects to antisociality). In particular, given the many personality traits and social factors that distinguish persons scoring high on a measure of antisocial behavior from other persons, what are possible reasons that these relationships are showing up as they are? For example, could extraversion (which can correlate with rule breaking or antisociality) be explaining the extent to which persons are leaving the house more often or standing closer to others – which would shift the implications of the findings in relation to risk factors and public health targets."

We agree with the importance of acknowledging other potential factors. Lines 450-457 in the limitations now discuss this point.

"I also noted that leaving home to go to work was also related to antisocial behavior, which again makes me wonder if there are potential social confounds or explanations that are not highlighted in explaining results. What could explain this connection?"…

This outcome was unexpected and has a variety of possible explanations, including the point above. For example, extraverted personality could tie together antisociality, social distancing and having a job that requires people to leave their home. Unfortunately, we do not have any additional data that could help explain this connection in any additional detail. We noted this point in our discussion of potential alternative explanations (lines 450-457). We also now highlight the association between socioeconomic variables and social distancing in the abstract (line 37-38).

Authors' responses to Reviewer #1:

"With regard to my second related concern, I think that the author's discussion in pages 398-402 should further clarify that their data seems to suggest at least two separate reasons for people leaving their home…"

We appreciate the reviewer's suggestion on how to clarify the results pertaining to subjects leaving for work. We have adapted the suggested wording to the manuscript (lines 400-407), but disagree with the framing of employment as a prosocial motivation—there are many reasons for going to work, only some of which would be described as prosocial.

"*…I would disagree with the statement in line 402 saying 'antisociality and reduced social distancing does not reflect these economic factors'…"*

We have edited the manuscript (lines 409-412) to reframe the interpretation. The new interpretation indicates that the relationship between antisociality and reduced social distancing persists when adjusting for socioeconomic factors that independently contributed to reduced social distancing.

"…I think that the authors should include in the manuscript the Chi square analyses showing that Black and Latino respondents left for home more frequently and had higher scores on the economic items of the STAB and discuss these findings beyond saying that the findings should not be interpreted to suggest that going to work is antisocial behavior… In a period in which there is significant need to understand and highlight the role that racial and ethnic inequities in American society play in various health outcomes that point must be made given the authors' data in this regard. Perhaps it should be synthesized with the discussion in lines 398-402/ the point I mentioned above."

We now include the statistic that Black and Hispanic participants left for work more frequently (lines 312-315), included this result in the discussion (lines 404-407), and added three relevant citations to large, population-level studies that have been recently published that measure and report racial and ethnic disparities related to COVID-19 (line 407, references 74-76). We are confident that with regards to this topic, readers will be best served by data from large studies that are designed and sufficiently powered to address it.

We are concerned about including data about the STAB economic items for Black and Hispanic participants, however. For transparency, this would require including and interpreting data from the rest of the STAB as well, and devoting a significant proportion of the manuscript to interpreting these analyses, which were not pre-registered, which were not hypothesized *a priori*, and which the study is underpowered for. Moreover, in examining the subsample of 26 Black (n=15) and/or Hispanic (n=15) participants, we determined they are younger (Black and Hispanic: M=32.96, SD=1.93; Non-Black or Hispanic: M=37.17, SD=0.98; t(129)=1.93, p=.056) and more likely to have a 4-yr degree (Black and Hispanic=81%; Non-Black or Hispanic=50%; χ^2=7.78, p=.005) than

the rest of the sample. The very small size of these samples, the non-hypothesized nature of differences as a function of race, and the non-equivalence of Black and Latino participants compared to other participants on key variables indicates to us that such analyses (which, again, our study was not designed for, and which were not preregistered) would be scientifically misguided and the results of such analyses uninterpretable. Worse, the results could be easily misconstrued in a way that negatively reflects on minority individuals, which would be a significant cost, particularly given the ambiguity of these data.
