# Supplementary material for: Reduced social distancing early in the COVID-19 pandemic is associated with antisocial behaviors in an online United States sample
Source: PLoS One. 2021 Jan 7;16(1):e0244974. doi: 10.1371/journal.pone.0244974 (PMC7790541; doi:10.1371/journal.pone.0244974)
Supplement: Attachment — Submitted filename: PLOSReview.docx [file pone.0244974.s002.docx]

The current study presents an analysis of 117 participants who completed self-report questionnaires regarding their COVID-19 pandemic-related compliance with social distancing and use of personal protective equipment (PPE, “mask, face shield and/or gloves”) in the previous week as well as basic demographic questions and the 32 item Antisocial Behavior Questionnaire (STAB) inquiring about antisocial behaviors across physical and social aggression, as well as rule breaking domains in the past year.

The authors concluded based on regression analyses including totals scores on the STAB and demographic variables that “antisociality predicted reduced social distancing during the COVID-19 pandemic.” The study has some strengths, including an interesting, novel approach to assess subjective social distancing and the authors should be commended for attempting to study of interesting variable in a very timely topic.

I have various concerns about the validity of the study’s conclusions that I outline below from major to minor below.

1) An examination of table 2 shows that the predictors for participants leaving their home in past week were “left for work” and “antisocial behavior” (i.e., STAB total score). The fact that “left for work” is as strong a predictor as “antisocial behavior” for leaving home was unaddressed by the authors and ignores a major alternative explanation for results of study: Economics. The authors may argue that because STAB total scores predicted leaving home in last week independently from “left for work” their conclusions stand. However, an examination of Table 4 indicates that “Rule-Breaking” subscale from the STAB was the most strongly related to “Times Left the House” and *the only* one to relate to “Distance Kept.” An examination of the items of the “Rule Breaking” scale from the STAB shows that there are three items that are all labor-related and could be endorsed by many people in their sample due to pandemic associate massive layoffs (”was suspended, expelled, or fired from school or work”; “had trouble keeping a job”; “failed to pay debts”). It would be interesting to see the rates of endorsement of items in the “Rule Breaking” subscale and see if after removing these 3 items from analyses the results would stand. It is very possible that the observed results for the “leaving home” outcome are driven by people who must leave for work because they are “essential” either in the higher end of education (e.g., medical personnel) but also in the lower end (cashiers, truckers, agricultural workers) who are also the most vulnerable to losing their jobs in the past year and thus endorse those 3 “Rule Breaking” items that basically penalize economic vicissitudes.
2) My hypothesis above is supported by the authors’ own data. Table 3 shows that less self- reported distance from others is predicted by *higher* education and *more* PPE use. In short, possibly by people who are essential workers in higher and lower SES strata who by the nature of their work (physician, cashier) *have* to leave home AND keep less distance from others. Again, the fact that *higher* education and PPE use predicts lower distance is glossed over and not addressed respectively in the discussion of the article, and supports the possibility that what we are seeing is the grim reality of the pandemic: Essential workers are having to put themselves at higher risk and the way they cope with is through increased PPE use. Those who have to leave home and be closer to others may do it because they have been “fired from work” and thus “had trouble keeping a job” and consequently are have “failed to pay debts.” Certainly not antisociality.
3) My hypothesis is further supported also by the authors’ own report that *none* of the STAB subscales or total score were related to PPE use. This further suggests that the reported relationship between “antisociality” and pandemic social distancing norms that the authors report have little to do with flaunting of social norms (mask wearing being one of the most contentious pandemic norms in the U.S.) and more with financial need to go to work. If antisociality assessed by the STAB which includes hitting, threatening, and swearing indeed was affecting these norms, some relation to PPE would be observed but that is not the case.
4) Incidentally, the data suggests that the contentiousness of social distancing and mask-wearing (or not) in the U.S. breaks along party/ideological identification with more democrat/liberal individuals endorsing more mask wearing whereas more republican/conservatives endorse less mask wearing (Pew Report, 2020). I don’t know if the authors asked any data in this respect but in general, republican/conservative individuals tend to endorse more traditional, norm following attitudes. Those data would militate against “antisocial behavior” as an explanation for failure to maintain social distance or remaining at home. The possibility that blue-collar jobs that are being threatened in this economy may be held by persons with more conservative ideology could however be playing a role. Or that more conservative leaders are disavowing social distancing and mask wearing citing economic reasons. Indeed, some of the qualitative data mentioned by the autors in p.18 suggest that economic anxiety is at the core of some of their participants’ behavior (“I worry that my retirement accounts won’t recover” “…cannot move ahead in life [sic]to the economy gets going again…”If the authors had a variable to address the influence of political ideology on COVID-19 related health norms would strengthen their study significantly. Alternatively, (although I am often reluctant to be the reviewer who says ‘collect more data’) doing another rapid survey on MTurk as the authors did, with a larger sample that assesses political ideology, recent loss of job because of the pandemic and economic stress due to it, and controls for it in their models could provide far more illuminating results.
5) An important variable related to the above concerns also not addressed in the analysis is the effect of ethnicity in their findings. The pandemic has hit lower SES workers and in especially Black and Latino workers and communities. The numbers of individuals from these communities are underrepresented in the study’s sample (perhaps another reason to continue collecting data and oversampling these populations) but nonetheless, it would behoove the authors to explore the interaction between these two ethnicities and endorsement of the three labor related items from the “Rule Breaking” scale of the STAB. The results may show yet another grim reality from the pandemic: The endorsement of having to leave home more often and be closer to other individuals is not due to “antisociality” but due to Black and Latino households having a higher rates of job losses during the pandemic, higher rates of burden for the caring of more family members, less work flexibility, and increased need to find more dangerous jobs during a pandemic.
6) Given the above concerns, the authors should explain why they chose to use the total score of the STAB-Total score in the regression analyses given that most of the variance is accounted for by the “rule breaking” scale. As I mentioned, I suspect that their results are better explained by endorsement of the 3 labor-related items in it and related to pandemic related massive layoffs rather than frank rule breaking. However, even if after removing these items, the (relatively) weak effect results hold, the authors would have to find a way to better contextualize their results. Otherwise they could be summarized as “people who do not follow strong social norms of behavior like stealing, and destroying private and public property are *more* likely to leave the home (paradoxically, possibly to work) and keep slightly less distance from others during a pandemic.” To be fair, the authors should be commended for trying to study such a fluid and novel phenomenon. However, the sample size, measures and robustness of analyses compares unfavorably with other efforts that are emerging with similar topic (e.g., Georgiou et al., 2020; Nowak et al., 2020).

Thank you for the opportunity to review this interesting manuscript.

References

Bartłomiej Nowak, Paweł Brzóska, Jarosław Piotrowski, Constantine Sedikides, Magdalena Żemojtel-Piotrowska, Peter K. Jonason, Adaptive and maladaptive behavior during the COVID-19 pandemic: The roles of Dark Triad traits, collective narcissism, and health beliefs, Personality and Individual Differences, Volume 167, 2020, 110232, ISSN 0191-8869, <https://doi.org/10.1016/j.paid.2020.110232>.

Neophytos Georgiou, Paul Delfabbro, Ryan Balzan, COVID-19-related conspiracy beliefs and their relationship with perceived stress and pre-existing conspiracy beliefs, Personality and Individual Differences, Volume 166, 2020, 110201, ISSN 0191-8869, <https://doi.org/10.1016/j.paid.2020.110201>.

“Republicans, Democrats Move Even Further Apart in Coronavirus Concerns.” Pew Research Center - U.S. Politics &amp; Policy, Pew Research Center, 24 July 2020.

www.pewresearch.org/politics/2020/06/25/republicans-democrats-move-even-further-apart-in-coronavirus-concerns/.
